# Effects of educational interventions for community pharmacists on promoting human papillomavirus vaccination: A randomized double-blind parallel group comparison trial

**DOI:** 10.1016/j.jvacx.2025.100607

**Published:** 2025-01-08

**Authors:** Nobuyuki Wakui, Mai Watanabe, Aika Okami, Hinako Kagi, Shoko Kawakubo, Yuna Hirota, Yui Onoda, Tomofumi Watanabe, Shunsuke Shirozu, Yoshiaki Machida, Mayumi Kikuchi

**Affiliations:** a Division of Applied Pharmaceutical Education and Research, Faculty of Pharmaceutical Sciences, Hoshi University, 2-4-41 Ebara, Shinagawa-ku, Tokyo 142-8501, Japan; b Shinagawa Pharmaceutical Association, 2-4-2 Nakanobu, Shinagawa-ku, Tokyo 142-0053, Japan

**Keywords:** HPV vaccination, Cervical Cancer prevention, Educational interventions, community pharmacists, Vaccine recommendation, Vaccination willingness

## Abstract

**Objective:**

Owing to persistent concerns about side effects, human papillomavirus (HPV) vaccination rates in Japan have remained low. Pharmacists are therefore encouraged to improve vaccination rates by providing accurate information. This study evaluated the impact of educational interventions on pharmacists' knowledge and willingness to recommend the HPV vaccine.

**Methods:**

This randomized double-blind trial assigned community pharmacists to an intervention or control group. The intervention group watched a video about the HPV vaccine and cervical cancer, whereas the control group watched a video about lung cancer. Assessments regarding knowledge and willingness to recommend the vaccine were conducted after obtaining consent (pre-test), immediately post-intervention (post-test 1), and 28 days post-intervention (post-test 2).

**Results:**

This study randomly assigned 124 participants. A significant difference in the change in motivation to recommend HPV vaccination at 28 days post-intervention (post-test 2) was observed between the two groups (*p* = 0.02). A significant difference in the change in motivation was also found between the two groups immediately post-intervention (post-test 1) (*p* < 0.001). Knowledge about the HPV vaccine and confidence in explaining it showed significant differences at both post-test 1 and post-test 2 (*p* < 0.001 for both).

**Discussion:**

Educational intervention significantly improved knowledge and willingness to recommend the HPV vaccine. This finding suggests that enhancing knowledge also boosts the willingness to recommend the vaccine, highlighting the potential long-term effects of educational interventions. Furthermore, our results underscore the crucial role pharmacists play in providing accurate information to the community.

**Conclusion:**

Utilizing pharmacists to disseminate vaccine information is effective given their familiarity with and accessibility to community residents. Policymakers should therefore leverage pharmacists to promote vaccine recommendations.

**Trial registration:**

UMIN Clinical Trials Registry Number: UMIN000050192. Registered Feb 23, 2023.

## Highlights

• The educational video increased pharmacists' motivation to recommend the HPV vaccine.
• Continuous support and clear communication are needed to sustain educational effects.
• Policymakers should strengthen pharmacists' roles in promoting HPV vaccination.

## 1 Introduction

Approximately 11,000 Japanese women are diagnosed with cervical cancer each year, while around 2900 die from it annually [1]. Most cases of cervical cancer are caused by the human papillomavirus (HPV), with HPV types 16 and 18 accounting for around 70 % of the cases [2]. The HPV vaccine, which prevents infection with HPV types 16 and 18 that cause cervical cancer [3], had been approved in Japan in 2009. By 2010, local governments began subsidizing vaccination costs. Furthermore, by April 2013, HPV vaccination had become routine for girls aged 12–16. However, in June of the same year, the Ministry of Health, Labour and Welfare suspended proactive recommendations for the vaccine due to concerns about side effects [4].

In contrast, the effectiveness of the HPV vaccine has been highly regarded overseas, with 2018 data showing vaccination rates exceeding 80 % in countries like Canada, the United Kingdom, and Australia [5,6]. However, following the suspension of proactive recommendations in Japan, the vaccination rate dropped significantly. In fact, among women born after 2002, the vaccination rate remained below 1 % [7]. Hence, despite the significant decrease in mortality rates from cervical cancer abroad, such rates have been on the rise in Japan [8].

The aim of the World Health Organization (WHO) is that by 2030, 90 % of females at the age of 15 would have been vaccinated against HPV [9]. Additionally, in 2017, the Japan Society of Obstetrics and Gynecology issued a statement calling for the early resumption of HPV vaccine recommendations to prevent an increase in cervical cancer among young women [10]. In response, Japan resumed proactive vaccine recommendations in April 2022 [11], following extensive studies that reaffirmed the HPV vaccine's safety [12]. To address the increasing incidence of cervical cancer linked to low vaccination rates during the suspension, the Ministry of Health, Labour and Welfare also introduced catch-up vaccinations for those who failed to receive the vaccine during this period [12]. All such efforts have been undertaken with the expectation that vaccination rates would begin to recover.

In Japan, excessive media coverage amplified concerns about side effects, leading to vaccine hesitancy and negative perceptions regarding the HPV vaccine. Despite being included in the National Immunization Program, the Ministry of Health, Labour and Welfare's withdrawal of its recommendation, based on limited scientific evidence, further eroded the public's trust in the vaccine [[13], [14], [15]]. Consequently, several healthcare professionals have been either hesitant about the HPV vaccine or lack sufficient knowledge about it. To improve vaccination rates, healthcare professionals must first possess accurate information about the vaccine and confidently convey this information while recommending vaccination. Healthcare providers' knowledge and attitudes toward the vaccine influence their delivery of a strong vaccine recommendation [16]. In particular, pharmacists, who are closely connected to the community, can play a significant role in increasing vaccination rates [17]. Studies abroad have also shown that pharmacists' extensive knowledge is associated with vaccine recommendations [18]. Additionally, research has demonstrated that convenient access to vaccination in pharmacies, along with strong professional recommendations, can significantly improve vaccination rates [[19], [20], [21]]. However, only a few pharmacists throughout Japan currently possess adequate knowledge about cervical cancer and HPV, leading to the inadequate provision of accurate information to the community [22].

Therefore, this study was planned based on the notion that improving pharmacists' knowledge might increase vaccination rates. This study aimed to investigate whether educational intervention about cervical cancer and the HPV vaccine can increase the number of pharmacists who intend to recommend the vaccine by enhancing their knowledge.

Specifically, we conducted a randomized double-blind parallel group comparison trial that divided pharmacists into two groups: those that viewed educational videos about the HPV vaccine and cervical cancer and those that did not. We then evaluated the changes in knowledge and willingness to recommend the vaccine through pre- and post-intervention surveys.

Through this study, we aim to improve pharmacists' knowledge and willingness to recommend the vaccine, thereby establishing a strategy to disseminate information to community residents through pharmacists.

## 2 Materials and methods

### 2.1 Study design

This randomized, double-blind, parallel-group comparison trial was conducted from February 13, 2023 to March 13, 2023. Participants were randomly assigned to either the intervention or control group. The intervention group viewed educational videos about the HPV vaccine and cervical cancer, whereas the control group viewed educational videos about lung cancer. The principal investigator explained the study's content and methods to all participants in advance, after which electronic informed consent was obtained from all participants. The study protocol was approved by the Ethics Committee for Research Involving Human Subjects at Hoshi University (Approval Number: 2022–02). Furthermore, this study was pre-registered with the University Hospital Medical Information Network Center (UMIN Trial ID: UMIN000050192).

### 2.2 Participants and setting

Participants were recruited using an email communication system targeting pharmacists who were members of the Shinagawa Ward Pharmacists Association in Tokyo. The inclusion criteria were as follows: (1) community pharmacists, (2) men and women aged 25–75, and (3) those who received a full explanation of the study's purpose and content, had the capacity to consent, fully understood the explanation, and voluntarily agreed to participate. The exclusion criteria were as follows: (1) those who refused to participate in the study of their own will and (2) those deemed ineligible by the principal investigator, including those for whom participation might cause undue burden or distress or who were unable to adhere to the study protocol.

### 2.3 Randomization and blinding

Participants were randomly assigned at a 1:1 ratio using the permuted block method with block sizes of 2 and 4. Randomization was carried out by an independent allocator to ensure unbiased assignment. The allocator created an assignment table with unique identification codes for each participant and sent YouTube URLs for the educational videos to maintain blinding. The intervention group received HPV-related videos, whereas the control group received smoking cessation videos. The assignment results were securely stored in a locked safe.

Intervention implementers and data analysts remained blinded until data analysis was complete. To ensure participant blinding, recruitment was performed under the title “Evaluation of the Educational Intervention Effect on Cancer for Pharmacists.” During the process of obtaining consent, participants were only informed that they would watch a cancer-related video and complete a questionnaire. This prevented the participants from knowing that the primary purpose of the intervention was related to cervical cancer and HPV. The study's main purpose and video contents were disclosed after all surveys were completed.

### 2.4 Creating the educational videos and questionnaires

The educational videos used for the intervention group, related to the HPV vaccine and cervical cancer, were created based on the “WHO Fact Sheet on Cervical Cancer” [9] and the “WHO Slides on the Elimination of Cervical Cancer as a Global Public Health Problem” (translated by the Japan Society of Obstetrics and Gynecology) [23]. On the other hand, the videos used for the control group, related to smoking, were edited for pharmacists from the “Slide Collection on Smoking Issues” [24] published by the Japan Lung Cancer Society for the general public.

The questionnaires were structured to prevent participants from identifying the primary cancer type targeted by the study, covering cervical cancer (Appendix Fig. 1), lung cancer (Appendix Fig. 2), and, as a dummy, liver cancer (Appendix Fig. 3). Participants answered all the questionnaires for the three cancer types during each testing session to maintain blinding. The number and format of questions for each type were designed to be similar. The cervical cancer questionnaire was based on survey items used in previous studies [25,26] to evaluate the effect of educational interventions, with additional or modified items to align with the current situation of HPV in Japan and the objectives of this study.

The lung cancer questionnaire was adjusted in terms of the number and content of questions to match the structure of the HPV vaccine questionnaire. Similarly, questions and content were set for liver cancer. The same questionnaire was used for the pre-test (i.e., before watching the video), post-test 1 (i.e., immediately after watching the video), and post-test 2 (i.e., 28 days after watching the video).

### 2.5 Pilot study

To verify the validity of the educational videos and questionnaires on the HPV vaccine and cervical cancer, as well as the overall trial schedule, a pilot study was conducted with the cooperation of 16 pharmacists working in insurance pharmacies, focusing only on the HPV vaccine group. The content of the videos was then revised based on feedback from the pharmacists who participated in the pilot study. Additionally, gynecologists were requested to review the content of the videos for validity, after which a final version of the videos for the main study was created, emphasizing the importance of both vaccination and regular screenings in the prevention of cervical cancer. The questionnaires were also validated by conducting a pre-test and two post-tests after watching the videos, following the trial schedule.

### 2.6 Procedures

The entire study lasted for 4 weeks, with the intervention consisting of video viewing within a single day. Participants completed a pre-test upon providing consent and registration. The intervention began at 10:00 AM on Day 1, during which all participants received an email simultaneously. This email contained the YouTube URL for the video and the URL for the web-based questionnaire via Google Forms. Participants were instructed to watch the video at any time during the specified period (Day 1 to Day 3) and complete post-test 1 immediately after viewing the video. Post-test 2 was conducted 28 days after viewing the video. Participants received another email with the URL for the web-based questionnaire via Google Forms and accessed this URL to complete post-test 2. Participants completed the questionnaire three times: (1) before watching the video, (2) immediately after watching the video, and (3) 28 days after watching the video. The questionnaire assessed their knowledge about cancer and its prevention methods, their willingness to recommend prevention methods, and their willingness to distribute and display leaflets and posters at their pharmacies.

### 2.7 Endpoint

The primary endpoint was the change from baseline in the willingness to recommend the HPV vaccine 28 days after the intervention. The secondary endpoints were the changes from baseline in the following aspects immediately after and 28 days after the educational intervention: (1) knowledge about the HPV vaccine, (2) confidence in explaining it to the patients, (3) willingness to display posters, and (4) willingness to distribute leaflets. Willingness and confidence were assessed using a 5-point Likert scale ranging from 1 (low) to 5 (high). Knowledge was assessed using a 10-item questionnaire, providing a score of 1 point for each correct answer and 0 point for each incorrect answer, with a total possible score of 10 points.

### 2.8 Statistical analysis

The obtained data were analyzed using SAS statistical software (version 9.4, SAS Institute, Inc., Cary, NC, USA). Our analysis population included participants who completed the video viewing and answered post-test 2, the primary endpoint, were analyzed. Google Forms was set to prevent the submission of questionnaires with any unanswered items, resulting in no missing values. Quantitative data were presented as means and standard deviations, whereas qualitative data were presented as frequencies and percentages. Each endpoint was analyzed using a mixed-effects model for repeated measures (MMRM), with baseline values as covariates and the changes from baseline at each time point as dependent variables. The effect size of the differences between groups for each endpoint was calculated using Cohen's d. All analyses were performed using two-sided tests, with *p* values of <0.05 indicating statistical significance.

## 3 Results

### 3.1 Participant information

**Fig. 1:**
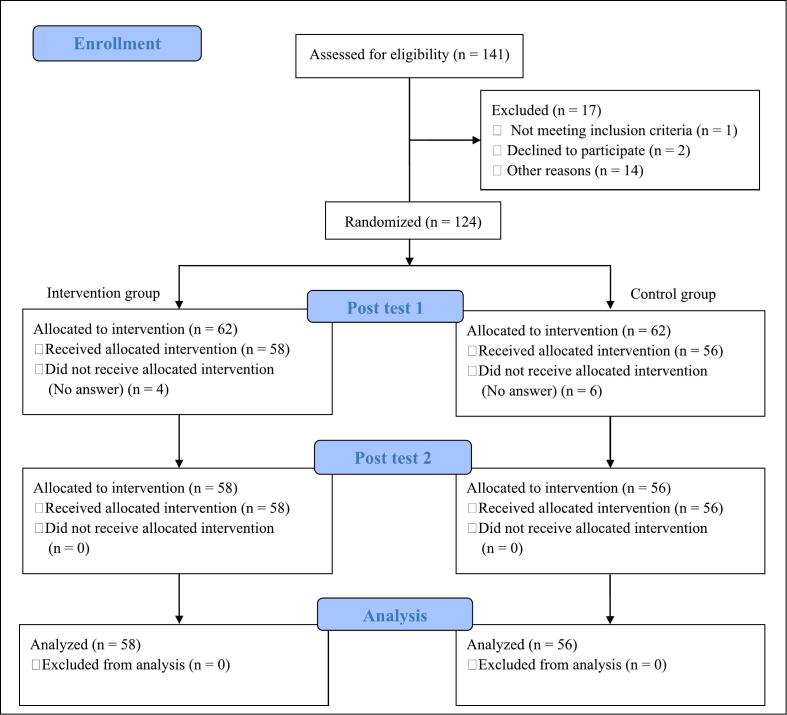
Flowchart of participant allocation.

A total of 141 pharmacists expressed interest in participating and were assessed for eligibility, with 17 pharmacists being subsequently excluded. Finally, 124 participants were included in the study and randomized (Fig. 1) into intervention and control group, each containing 62 participants. Four participants in the intervention group and six in the control group did not complete post-test 1, resulting in 58 completers in the intervention group and 56 in the control group, for a total of 114 completers. These 114 participants also completed post-test 2 conducted 28 days after the intervention and were included in the analysis population.

Participants in both groups had similar baseline characteristics (Table 1). The mean age of all participants was 42.8 ± 10.8 years, and 60.5 % were female. A large proportion (93.9 %) of the pharmacies wherein the participants worked accommodated cancer patients. The pre-test results showed that knowledge, willingness to recommend preventive methods, willingness to distribute and display leaflets and posters, and responses to questions about the three types of cancer were similar between the two groups.

**Table 1:** Participant characteristics at baseline.

	Intervention group (*n* = 58)	Control group (*n* = 56)	Total (*n* = 114)
Age	42.9 ± 10.7	42.8 ± 11.0	42.8 ± 10.8
Sex (Female)	34 (58.6 %)	35 (62.5 %)	69 (60.5 %)
Do cancer patients ever come to the pharmacy where you work?	56 (96.6 %)	51 (91.1 %)	107 (93.9 %)

Pre-test			
Cervical cancer			
Knowledge of Cervial cancer and HPV vaccine	5.53 ± 2.36	5.55 ± 2.09	5.54 ± 2.22
Do you want to recommend the HPV vaccine to your patients?	3.83 ± 0.84	3.77 ± 0.79	3.80 ± 0.81
How confident are you in your ability to adequately explain the HPV vaccine to patients who are considering it?	2.07 ± 0.86	2.07 ± 0.95	2.07 ± 0.90
Do you want to display a poster about cervical cancer and its prevention in your pharmacy?	3.84 ± 1.11	3.82 ± 1.01	3.83 ± 1.06
Do you want to distribute leaflets about cervical cancer and its prevention in your pharmacy?	3.86 ± 1.03	3.89 ± 0.89	3.88 ± 0.96

Lung cancer			
Knowledge of Lung cancer and Tobacco	7.33 ± 1.26	7.61 ± 1.15	7.46 ± 1.21
Do you want to recommend quitting smoking to your patients?	4.69 ± 0.47	4.36 ± 0.98	4.53 ± 0.78
How confident are you in your ability to adequately explain the benefits of quitting smoking to patients who are considering it?	3.09 ± 0.94	3.11 ± 1.06	3.10 ± 1.00
Do you want to display a poster about lung cancer and its prevention in the pharmacy?	4.05 ± 0.93	4.04 ± 1.01	4.04 ± 0.96
Do you want to distribute leaflets about lung cancer and its prevention in your pharmacy?	4.10 ± 0.79	4.02 ± 0.75	4.06 ± 0.77

Liver cancer			
Knowledge of Liver cancer and hepatitis virus	6.12 ± 1.79	6.57 ± 1.98	6.34 ± 1.89
Do you want to recommend the hepatitis B virus vaccine to patients?	3.84 ± 0.81	3.79 ± 0.97	3.82 ± 0.89
How confident are you in your ability to properly explain the hepatitis B virus vaccination to patients considering it?	2.10 ± 0.99	2.13 ± 1.01	2.11 ± 0.99
Do you want to display a poster about liver cancer and its prevention in your pharmacy?	3.98 ± 0.93	3.91 ± 1.00	3.95 ± 0.96
Do you want to distribute leaflets about liver cancer and its prevention in your pharmacy?	4.07 ± 0.79	3.96 ± 0.76	4.02 ± 0.78

### 3.2 Results after educational intervention

The results of post-test 1 and post-test 2 on cervical cancer after the educational intervention are summarized in Table 2. The intervention group showed higher scores than the non-intervention group on all items at each time point. In the HPV group, the total scores for post-test 1 immediately after the intervention were higher than those for post-test 2 conducted 28 days after the intervention, indicating a tendency for scores to decrease over time. In contrast, the non-intervention group showed somewhat higher scores in post-test 2 than in post-test 1, except for items related to the willingness to recommend the HPV vaccine to patients.

**Table 2:** Survey results on cervical cancer after the educational intervention.

	Intervention group (*n* = 58)		Control group (n = 56)	
	Post test 1	Post test 2	Post test 1	Post test 2
Cervical cancer				
Knowledge of Cervial cancer and HPV vaccine	8.97 ± 0.92	8.60 ± 1.21	6.48 ± 1.80	6.91 ± 1.80
Do you want to recommend the HPV vaccine to your patients?	4.29 ± 0.96	4.12 ± 0.96	3.77 ± 0.69	3.71 ± 0.91
How confident are you in your ability to adequately explain the HPV vaccine to patients who are considering it?	3.64 ± 0.72	3.23 ± 0.83	2.48 ± 0.99	2.54 ± 0.99
Do you want to display a poster about cervical cancer and its prevention in your pharmacy?	4.22 ± 0.86	4.10 ± 0.89	3.77 ± 1.01	3.91 ± 0.94
Do you want to distribute leaflets about cervical cancer and its prevention in your pharmacy?	4.33 ± 0.78	4.09 ± 0.84	3.83 ± 0.93	3.91 ± 0.86

### 3.3 Changes in motivation to recommend HPV vaccination

After comparing the motivation to recommend HPV vaccination between the two groups 28 days after the intervention (Table 3), we found that the least squares mean difference in the change from baseline was 0.38 (95 % CI: 0.05–0.71), indicating a significant difference (*p* = 0.02, effect size: 0.43). Additionally, immediately after the intervention, the least squares mean difference in the change from baseline for motivation to recommend HPV vaccination was 0.50 (95 % CI: 0.22–0.78), also indicating a significant difference (*p* < 0.001, effect size: 0.66).

**Table 3:** Changes in motivation to recommend HPV vaccination after educational intervention.

	LS-Means (95 % CI)		Differences of LSMD	*p*-value	effect size
	Intervention group (n = 58)	Control group (n = 56)			
Willingness to recommend HPV vaccination					
Post test 1	0.48 (0.29 to 0.68)	−0.02 (−0.22 to 0.18)	0.50 (0.22 to 0.78)	<0.001	0.66
Post test 2	0.31 (0.08 to 0.54)	−0.07 (−0.31 to 0.16)	0.38 (0.05 to 0.71)	0.02	0.43

The question “Do you want to recommend the HPV vaccine to your patients?” was shortened to “Willingness to recommend HPV vaccination.”, LSMD: least squares mean difference., 95 % CI: 95 % confidence interval.

### 3.4 Knowledge and confidence in explaining HPV vaccination to patients

The least squares mean difference in knowledge about the HPV vaccine and confidence in explaining it differed significantly between the two groups at all time points (Table 4). The effect sizes for both items were larger at post-test 1 than at post-test 2.

**Table 4:** Changes in knowledge and confidence in explaining HPV vaccination to patients after the educational intervention.

	LS-Means (95 % CI)		Differences of LSMD	p-value	effect size
	Intervention group (n = 58)	Control group (n = 56)			
Post test 1					
Knowledge about the HPV vaccine	3.42 (3.10 to 3.74)	0.94 (0.61 to 1.26)	2.49 (2.03 to 2.95)	<0.001	2.02
Confidence in explaining properly	1.57 (1.39 to 1.75)	0.41 (0.23 to 0.60)	1.16 (0.90 to 1.42)	<0.001	1.65

Post test 2					
Knowledge about the HPV vaccine	3.06 (2.72 to 3.40)	1.36 (1.02 to 1.71)	1.70 (1.21 to 2.19)	<0.001	1.30
Confidence in explaining properly	1.19 (0.99 to 1.39)	0.46 (0.26 to 0.67)	0.72 (0.43 to 1.01)	<0.001	0.93

The question “How confident are you that you can properly explain HPV vaccination to patients considering HPV vaccination?” was shortened to “Confidence in explaining properly.”, LSMD: least squares mean difference., 95 % CI: 95 % confidence interval.

### 3.5 Changes in willingness to display posters and distribute leaflets about the HPV vaccine

The least squares mean difference in willingness to display posters about the HPV vaccine and distribute leaflets between two groups 28 days after the intervention was 0.18 (95 % CI: −0.06–0.42; *p* = 0.14) for posters and 0.19 (95 % CI: −0.04–0.43; *p* = 0.11) for leaflets. A comparison between the intervention and control groups revealed significant differences at post-test 1; however, no significant differences in the willingness to display posters about the HPV vaccine and distribute leaflets were found at post-test 2 (Table 5).

**Table 5:** Changes in the willingness to display posters and distribute leaflets about the HPV vaccine after the educational intervention.

	LS-Means (95 % CI)		Differences of LSMD	p-value	effect size
	Intervention group (n = 58)	Control group (n = 56)			
**Post test 1**					
Willingness to display posters	0.38 (0.20 to 0.57)	−0.06 (−0.25 to 0.13)	0.44 (0.18 to 0.71)	0.001	0.62
Willingness to distribute leaflets	0.46 (0.28 to 0.64)	−0.05 (−0.23 to 0.14)	0.51 (0.25 to 0.76)	<0.001	0.73

**Post test 2**					
Willingness to display posters	0.26 (0.09 to 0.43)	0.08 (−0.09 to 0.26)	0.18 (−0.06 to 0.42)	0.14	0.28
Willingness to distribute leaflets	0.22 (0.05 to 0.38)	0.02 (−0.14 to 0.19)	0.19 (−0.04 to 0.43)	0.11	0.30

The question “Do you want to display a poster about cervical cancer and its prevention in the pharmacy?” was shortened to “Willingness to display posters,” while the question “Do you want to distribute leaflets about cervical cancer and its prevention in your pharmacy?” was shortened to “Confidence in explaining properly.”, LSMD: least squares mean difference., 95 % CI: 95 % confidence interval.

## 4 Discussion

The current study randomly divided 124 pharmacists into an intervention and a control group to ensure a fair comparison. Pharmacists who received the educational intervention on cervical cancer and the HPV vaccine showed significantly improved knowledge and willingness to recommend the vaccine, even after 28 days. These findings suggest that educating pharmacists boosts not only their knowledge but also their long-term commitment to promoting vaccination.

Significant changes in the willingness to recommend HPV vaccination were observed both immediately after the educational intervention and 28 days thereafter. The sustained difference until 28 days post-intervention highlights the long-term impact of educating pharmacists about vaccination. The immediate improvement in knowledge about cervical cancer and the HPV vaccine also increased willingness to recommend the vaccine. Previous research has shown that pharmacists with higher knowledge levels are more likely to recommend vaccination [18]. Knowledge, Attitude, and Practice surveys have also found that knowledge and attitudes significantly influence vaccination willingness among various groups like students [[27], [28], [29]]. In Japan, numerous community pharmacists engage with their communities through health education and consultations, with some also serving as school pharmacists who provide lectures on medications at local elementary and middle schools [30,31]. Hence, equipping such pharmacists with accurate knowledge and strengthening their motivation to recommend the HPV vaccine can effectively promote HPV awareness among students and parents, leading to increased vaccination rates in their communities.

Japan has over 60,000 pharmacies that can serve as an ideal platform for disseminating health information, such as promoting HPV vaccination through posters and leaflets. Our study found that pharmacists' willingness to display these materials significantly increased immediately after viewing the educational video. However, a slight decline in such willingness was observed after 28 days, suggesting a decrease in the impact of initially effective educational interventions over time without ongoing reinforcement. Although the Japanese government and pharmaceutical companies currently provide these materials to pharmacies, simply distributing them is not enough. Clear communication of the purpose of these materials and provision of continuous educational support to maintain pharmacists' motivation are essential. The WHO has highlighted the critical role of community pharmacists as the most accessible healthcare professionals, forming a foundation for primary healthcare [32]. Consistent with Japan's policy to transform pharmacies into health support stations [33], implementation of specific support measures that sustain pharmacists' engagement in these efforts over time is crucial. Ensuring that pharmacies effectively serve this role will require systematic frameworks and continuous support, making these initiatives more meaningful and impactful for local residents.

The current study has the following two limitations. First, it remains unclear whether the pharmacists who received the educational intervention actually provided information to local residents. Second, considering that the same questionnaire was administered to the subjects three times (one pre-test and two post-tests), the non-intervention group could have possibly learned about HPV from the questions, which may have reduced the difference in knowledge between the intervention and control groups. Despite these limitations, this study remains significant and valuable as it employed a double-blind comparative design to investigate the effects of educational interventions on cervical cancer and the HPV vaccine for community pharmacists through Japan where HPV vaccination rates are extremely low compared to that worldwide. Overall, the current study highlights the effectiveness of educational interventions, making it a highly significant and valuable study.

Another significant aspect of this study is that it demonstrated the increased and sustained willingness of community pharmacists to recommend HPV vaccination following an educational intervention and highlighted the usefulness of utilizing pharmacists—healthcare professionals who are readily accessible to local residents—as providers of information. Studies abroad have shown that educational activities can significantly enhance the general public's knowledge of the HPV vaccine and their intent to get vaccinated [[34], [35], [36]]. Although these observational studies demonstrated associations, our randomized double-blind controlled trial provides strong evidence that targeted educational programs can directly boost knowledge and proactive engagement among healthcare professionals, reinforcing their vital role in public health initiatives. A randomized controlled trial involving children and their parents overseas also underscored the effectiveness of viewing videos that summarize the benefits and risks of the HPV vaccine [37]. Similarly, the educational videos viewed by community pharmacists in our study increased not only their knowledge but also their willingness to recommend the vaccine, suggesting that video-based education is an effective tool for promoting vaccination.

The educational interventions implemented herein could play a crucial role in addressing barriers such as vaccine hesitancy and lack of awareness, with many people either not feeling the need for the HPV vaccine or being unaware of its existence. By offering residents reliable information from trusted healthcare professionals, these interventions create opportunities for local residents to receive appropriate guidance, which is expected to increase vaccination rates. Our educational intervention demonstrated the possibility of enhancing pharmacists' knowledge about the HPV vaccine and their willingness to recommend it, suggesting that pharmacies can effectively support the vaccination of local residents. Based on the results presented herein, policymakers should recognize and leverage the value of pharmacists as healthcare professionals to promote vaccination and provide accurate information to the community.

## 5 Conclusion

This study demonstrated that educating pharmacists about cervical cancer and the HPV vaccine improved not only their knowledge but also their willingness to recommend the HPV vaccine. Utilizing pharmacists, who are familiar with and accessible to community residents, to disseminate vaccine information is highly effective. Policymakers should leverage the trusted position of pharmacists to promote vaccination and improve public health outcomes.

## Funding

This work was supported by 10.13039/501100001691JSPS KAKENHI Grant Number JP23K06289 and a research grant from the Japanese Society of Pharmaceutical Oncology. The findings and conclusions contained within are those of the authors and do not necessarily reflect the positions or policies of the funding agencies.

## CRediT authorship contribution statement

**Nobuyuki Wakui:** Writing – review & editing, Writing – original draft, Supervision, Project administration, Methodology, Investigation, Funding acquisition, Formal analysis, Conceptualization. **Mai Watanabe:** Writing – review & editing, Writing – original draft, Methodology, Investigation, Formal analysis, Conceptualization. **Aika Okami:** Investigation. **Hinako Kagi:** Investigation. **Shoko Kawakubo:** Investigation. **Yuna Hirota:** Investigation. **Yui Onoda:** Investigation. **Tomofumi Watanabe:** Writing – review & editing. **Shunsuke Shirozu:** Writing – review & editing, Data curation. **Yoshiaki Machida:** Writing – review & editing. **Mayumi Kikuchi:** Writing – review & editing, Investigation.

## Declaration of competing interest

The authors declare that they have no known competing financial interests or personal relationships that could have appeared to influence the work reported in this paper.

## Data Availability

Data will be made available on request.

## Appendix A Supplementary data

Supplementary material 1 Image 1

Supplementary material 2 Image 2
