# Supplementary material for: Effects of educational interventions for community pharmacists on promoting human papillomavirus vaccination: A randomized double-blind parallel group comparison trial
Source: Vaccine X. 2025 Jan 8;22:100607. doi: 10.1016/j.jvacx.2025.100607 (PMC11774807; doi:10.1016/j.jvacx.2025.100607)
Supplement: Supplementary file 2 — Supplementary material 2 [file mmc2.docx]

Appendix Figure.1 The questionnaires of Cervical cancer

| **Cervical cancer** | | | | |
| --- | --- | --- | --- | --- |
| This section contains questions about cervical cancer. For Q1–Q9: Select 'Yes,' 'No,' or 'I don't know' if you are unsure or have difficulty deciding. For Q10: Select 'Yes' or 'No' for each item. For Q11–Q14: Select the option that best reflects your response. | | | | |
| Q1 | Do you think HPV infection is rare? | ○ | Yes | |
|  |  | ○ | No | |
|  |  | ○ | I don't know | |
| Q2 | Do you think HPV is a sexually transmitted infection (STI)? | ○ | Yes | |
|  |  | ○ | No | |
|  |  | ○ | I don't know | |
| Q3 | Do you think HPV can sometimes cause cancer? | ○ | Yes | |
|  |  | ○ | No | |
|  |  | ○ | I don't know | |
| Q4 | Do you think HPV can sometimes cause oral cancer? | ○ | Yes | |
|  |  | ○ | No | |
|  |  | ○ | I don't know | |
| Q5 | Do you think HPV can go away on its own without treatment? | ○ | Yes | |
|  |  | ○ | No | |
|  |  | ○ | I don't know | |
| Q6 | Do you think HPV is preventable? | ○ | Yes | |
|  |  | ○ | No | |
|  |  | ○ | I don't know | |
| Q7 | Do you think HPV can cause genital warts? | ○ | Yes | |
|  |  | ○ | No | |
|  |  | ○ | I don't know | |
| Q8 | Do you think the HPV vaccine can be administered to girls? | ○ | Yes | |
|  |  | ○ | No | |
|  |  | ○ | I don't know | |
| Q9 | Do you think the HPV vaccine can be administered to boys? | ○ | Yes | |
|  |  | ○ | No | |
|  |  | ○ | I don't know | |
| Q10 | Which of the following do you think the HPV vaccine can reduce or prevent? |  | Yes | No |
|  |  | Cervical cancer | ○ | ○ |
|  |  | Chlamydia | ○ | ○ |
|  |  | Genital warts | ○ | ○ |
|  |  | Head and neck cancer | ○ | ○ |
|  |  | HIV | ○ | ○ |
| Q11 | Do you want to recommend the HPV vaccine to your patients? | ○ | Do not recommend | |
|  |  | ○ | Somewhat don't recommend | |
|  |  | ○ | Neutral | |
|  |  | ○ | Somewhat recommend | |
|  |  | ○ | Recommend | |
| Q12 | How confident are you in your ability to adequately explain the HPV vaccine to patients who are considering it? | ○ | Not confident at all | |
|  |  | ○ | Somewhat not confident | |
|  |  | ○ | Neutral | |
|  |  | ○ | Somewhat confident | |
|  |  | ○ | Very confident | |
| Q13 | Do you want to display a poster about cervical cancer and its prevention in your pharmacy? | ○ | Strongly disagree | |
|  |  | ○ | Somewhat disagree | |
|  |  | ○ | Neutral | |
|  |  | ○ | Somewhat agree | |
|  |  | ○ | Strongly agree | |
| Q14 | Do you want to distribute leaflets about cervical cancer and its prevention in your pharmacy? | ○ | Strongly disagree | |
|  |  | ○ | Somewhat disagree | |
|  |  | ○ | Neutral | |
|  |  | ○ | Somewhat agree | |
|  |  | ○ | Strongly agree | |

Appendix Figure.2 The questionnaires of Lung cancer

| **Lung cancer** | | | | |
| --- | --- | --- | --- | --- |
| This section contains questions about lung cancer. For Q1–Q9: Select 'Yes,' 'No,' or 'I don't know' if you are unsure or have difficulty deciding. For Q10: Select 'Yes' or 'No' for each item. For Q11–Q14: Select the option that best reflects your response. | | | | |
| Q1 | Do you think smoking causes lung cancer? | ○ | Yes | |
|  |  | ○ | No | |
|  |  | ○ | I don't know | |
| Q2 | Do you think smoking causes heart attacks (myocardial infarction)? | ○ | Yes | |
|  |  | ○ | No | |
|  |  | ○ | I don't know | |
| Q3 | Do you think the number of deaths from lung cancer is increasing? | ○ | Yes | |
|  |  | ○ | No | |
|  |  | ○ | I don't know | |
| Q4 | Do you think asbestos is greater risk factor for lung cancer than smoking? | ○ | Yes | |
|  |  | ○ | No | |
|  |  | ○ | I don't know | |
| Q5 | Do you think secondhand smoke causes lung cancer? | ○ | Yes | |
|  |  | ○ | No | |
|  |  | ○ | I don't know | |
| Q6 | Do you think that someone who smoked in the past but no longer smokes has the same risk for lung cancer as a non-smoker? | ○ | Yes | |
|  |  | ○ | No | |
|  |  | ○ | I don't know | |
| Q7 | Do you think secondhand smoke affects children's intelligence? | ○ | Yes | |
|  |  | ○ | No | |
|  |  | ○ | I don't know | |
| Q8 | Do you think alcohol consumption has a greater impact on health than smoking? | ○ | Yes | |
|  |  | ○ | No | |
|  |  | ○ | I don't know | |
| Q9 | Do you think smoking affects life expectancy? | ○ | Yes | |
|  |  | ○ | No | |
|  |  | ○ | I don't know | |
| Q10 | After how many years of quitting smoking do you think the risk of lung cancer becomes the same as that of a non-smoker? |  | Yes | No |
|  |  | 10 years | ○ | ○ |
|  |  | 20 years | ○ | ○ |
|  |  | 30 years | ○ | ○ |
|  |  | 40 years | ○ | ○ |
|  |  | 50 years | ○ | ○ |
| Q11 | Do you want to recommend quitting smoking to your patients? | ○ | Do not recommend | |
|  |  | ○ | Somewhat don't recommend | |
|  |  | ○ | Neutral | |
|  |  | ○ | Somewhat recommend | |
|  |  | ○ | Recommend | |
| Q12 | How confident are you in your ability to adequately explain the benefits of quitting smoking to patients who are considering it? | ○ | Not confident at all | |
|  |  | ○ | Somewhat not confident | |
|  |  | ○ | Neutral | |
|  |  | ○ | Somewhat confident | |
|  |  | ○ | Very confident | |
| Q13 | Do you want to display a poster about lung cancer and its prevention in the pharmacy? | ○ | Strongly disagree | |
|  |  | ○ | Somewhat disagree | |
|  |  | ○ | Neutral | |
|  |  | ○ | Somewhat agree | |
|  |  | ○ | Strongly agree | |
| Q14 | Do you want to distribute leaflets about lung cancer and its prevention in your pharmacy? | ○ | Strongly disagree | |
|  |  | ○ | Somewhat disagree | |
|  |  | ○ | Neutral | |
|  |  | ○ | Somewhat agree | |
|  |  | ○ | Strongly agree | |

Appendix Figure.3 The questionnaires of Liver cancer

| **Liver cancer** | | | | |
| --- | --- | --- | --- | --- |
| This section contains questions about liver cancer. For Q1–Q9: Select 'Yes,' 'No,' or 'I don't know' if you are unsure or have difficulty deciding. For Q10: Select 'Yes' or 'No' for each item. For Q11–Q14: Select the option that best reflects your response. | | | | |
| Q1 | Do you think the hepatitis B virus causes hepatocellular carcinoma? | ○ | Yes | |
|  |  | ○ | No | |
|  |  | ○ | I don't know | |
| Q2 | Do you think the hepatitis C virus causes hepatocellular carcinoma? | ○ | Yes | |
|  |  | ○ | No | |
|  |  | ○ | I don't know | |
| Q3 | Do you think the hepatitis B virus causes cirrhosis? | ○ | Yes | |
|  |  | ○ | No | |
|  |  | ○ | I don't know | |
| Q4 | Do you think a vaccine can prevent hepatitis B virus infection? | ○ | Yes | |
|  |  | ○ | No | |
|  |  | ○ | I don't know | |
| Q5 | Do you think a vaccine can prevent hepatitis C virus infection? | ○ | Yes | |
|  |  | ○ | No | |
|  |  | ○ | I don't know | |
| Q6 | Do you think the hepatitis B virus vaccine is part of the routine immunization schedule? | ○ | Yes | |
|  |  | ○ | No | |
|  |  | ○ | I don't know | |
| Q7 | Do you think the hepatitis B virus vaccine can be administered to adults? | ○ | Yes | |
|  |  | ○ | No | |
|  |  | ○ | I don't know | |
| Q8 | Do you think there are countries where the hepatitis B virus vaccine is administered to the entire population? | ○ | Yes | |
|  |  | ○ | No | |
|  |  | ○ | I don't know | |
| Q9 | Do you think that people infected with the hepatitis virus will inevitably develop hepatocellular carcinoma? | ○ | Yes | |
|  |  | ○ | No | |
|  |  | ○ | I don't know | |
| Q10 | How many people in the country do you think are infected with the hepatitis B virus? |  | Yes | No |
|  |  | 1 in 10 people | ○ | ○ |
|  |  | 1 in 100 people | ○ | ○ |
|  |  | 1 in 1,000 people | ○ | ○ |
|  |  | 1 in 10,000 people | ○ | ○ |
|  |  | 1 in 100,000 people | ○ | ○ |
| Q11 | Do you want to recommend the hepatitis B virus vaccine to patients? | ○ | Do not recommend | |
|  |  | ○ | Somewhat don't recommend | |
|  |  | ○ | Neutral | |
|  |  | ○ | Somewhat recommend | |
|  |  | ○ | Recommend | |
| Q12 | How confident are you in your ability to properly explain the hepatitis B virus vaccination to patients considering it? | ○ | Not confident at all | |
|  |  | ○ | Somewhat not confident | |
|  |  | ○ | Neutral | |
|  |  | ○ | Somewhat confident | |
|  |  | ○ | Very confident | |
| Q13 | Do you want to display a poster about liver cancer and its prevention in your pharmacy? | ○ | Strongly disagree | |
|  |  | ○ | Somewhat disagree | |
|  |  | ○ | Neutral | |
|  |  | ○ | Somewhat agree | |
|  |  | ○ | Strongly agree | |
| Q14 | Do you want to distribute leaflets about liver cancer and its prevention in your pharmacy? | ○ | Strongly disagree | |
|  |  | ○ | Somewhat disagree | |
|  |  | ○ | Neutral | |
|  |  | ○ | Somewhat agree | |
|  |  | ○ | Strongly agree | |
